# Postpartum Depressive Symptoms and Mother–Infant Bonding at 4–24 Weeks Postpartum: A Cross-Sectional Study of Psychosocial and Breastfeeding-Related Factors

**DOI:** 10.3390/jcm15218677

**Published:** 2026-11-06

**Authors:** Elif Küçük, Mehmet Tolga Köle, Deniz Güven, Seval Öztürk, İbrahim Kandemir, Ezgi Özserezli, Feride Uysal, Burak Ersak, Selvihan Yılmaz Özdemir, Emel Örün

**Affiliations:** 1 Department of Psychiatry, Faculty of Medicine, Yüksek İhtisas University, 06530 Ankara, Türkiye; drelfkucuk@gmail.com; 2 Department of Pediatrics, Etlik City Hospital, 06170 Ankara, Türkiye; deniz.guven06@hotmail.com (D.G.); s.ozturkd@gmail.com (S.Ö.); drselvihan@gmail.com (S.Y.Ö.); emelorun@hotmail.com (E.Ö.); 3 Department of Pediatrics, Istanbul Health and Technology University, 34445 İstanbul, Türkiye; dr.ibrahimkandemir@gmail.com; 4 Department of Psychiatry, Etlik City Hospital, 06170 Ankara, Türkiye; ezgi_cetinel@hotmail.com; 5 Department of Psychiatry, Kartal Dr. Lutfi Kirdar City Hospital, 34865 İstanbul, Türkiye; drferidekeskinler@gmail.com; 6 Department of Gynecologic Oncology, Etlik City Hospital, 06170 Ankara, Türkiye; burakersak@gmail.com

**Keywords:** postpartum depression, mother–infant bonding, breastfeeding, partner support, pregnancy

## Abstract

**Background/Objectives**: Postpartum depressive symptoms have been associated with maternal functioning and mother–infant bonding (MIB). This study primarily examined the association between postpartum depressive symptom severity and MIB, estimated the proportion screening positive for probable postpartum depression (PPD), and explored relevant sociodemographic and psychosocial correlates. **Methods:** In this cross-sectional study, 212 mothers assessed at 4–24 weeks postpartum during April–June 2025 completed the Edinburgh Postnatal Depression Scale (EPDS) and Maternal Attachment Inventory (MAI). The primary adjusted analysis used linear regression with HC3 robust standard errors, including infant age and selected clinical and psychosocial covariates. Because 39.2% of MAI scores were at the maximum, Tobit, fractional-logit, and ordinal regressions were used as complementary sensitivity analyses, alongside exploratory spline assessments. **Results**: Forty-one mothers (19.3%) screened positive for probable PPD (EPDS ≥13). Each 1-point higher EPDS score was associated with a 0.20-point lower MAI score (95% CI: −0.26 to −0.15), approximately one MAI point per five EPDS points. Observed-scale estimates were similar across linear, Tobit, and fractional-logit models, although exploratory splines suggested possible nonlinearity at sparsely represented EPDS extremes. In exploratory adjusted analyses, stressful breastfeeding and nonbreastfeeding were associated with higher EPDS scores. Separately, only four of 69 unadjusted exploratory comparisons survived false-discovery-rate correction, all involving probable PPD screening status and breastfeeding or spousal support. **Conclusions**: Greater depressive symptom severity was associated with lower bonding scores, but the absolute difference was small and its clinical significance uncertain. The cross-sectional design precludes causal inference.

## 1. Introduction

Pregnancy and the months after birth bring substantial biological and psychosocial change, and with it a heightened vulnerability to mental health problems [1]. The conditions women experience in this period span a wide range, from transient postpartum blues through postpartum depression (PPD) to severe presentations such as postpartum psychosis [2]. PPD is among the most common complications of childbirth, affecting approximately 19% of women worldwide, although national estimates range from about 3% to 44% [3]. Rates tend to be higher in low- and middle-income countries than in high-income ones, so this burden falls unevenly along socioeconomic and geographical lines [4,5]. Symptoms may first appear at any point during the first postpartum year, and longitudinal evidence shows that in some women they persist into the second [6].

The determinants of PPD are multifactorial, spanning sociodemographic, psychosocial, and maternal health domains. Low socioeconomic status, inadequate partner and social support, unplanned pregnancy, adverse living conditions, and complications of pregnancy or the postpartum period have each been associated with increased risk [7,8]. The underlying evidence base is substantial: a global meta-analysis of 565 studies and 1,236,365 women linked PPD to marital and educational status, economic hardship, life stress, pregnancy planning, partner relationships, exposure to violence, breastfeeding, and living conditions [4]. Adolescent motherhood adds a further layer of vulnerability. Reviews have reported postpartum depression rates roughly twice those of adult mothers, at around 25% [9,10], and breastfeeding initiation appears less common among younger mothers: 44% among adolescent mothers versus 65% among older mothers in a large US cohort [11]. These estimates derive from specific populations and may not generalize, but they point to age-related differences in both postpartum mental health and infant feeding. Relational factors have also been linked to bonding itself. Low perceived social support, self-criticism, and insecure adult attachment have been associated with poorer mother–infant bonding (MIB) where PPD symptoms are present [12,13], whereas partner support and secure attachment have been associated with fewer depressive symptoms and more favorable bonding outcomes [14,15].

Postpartum depressive symptoms have been associated with bonding itself and with maternal–infant interaction, breastfeeding practices, and caregiving behavior [16,17]. MIB refers to the emotional connection that develops between a mother and her infant after birth, although related bonding processes may begin during pregnancy [18]. Weaker bonding, in turn, has been linked to less favorable infant attachment-related outcomes and to regulatory difficulties such as excessive crying and sleep problems [19,20]. Perinatal depressive and anxiety symptoms have themselves been associated with poorer offspring development across socioemotional, cognitive, language, motor, and adaptive domains, with some associations extending into childhood and adolescence [21,22,23].

In Türkiye, Örün et al. examined depressive symptoms and mother–infant bonding at two months postpartum [24], while Çankaya et al. and Topbas Selcuki et al. studied depressive symptoms and maternal attachment in other postpartum samples [25,26]. The present study differs in two respects. First, mothers were assessed across a wider window, at varying points between 4 and 24 weeks postpartum, rather than at a single time point. Second, the adjusted association between depressive symptom severity and bonding was examined using complementary regression models, chosen to assess how far the pronounced MAI ceiling effect shaped the estimate. The proportion screening positive for probable PPD was a secondary descriptive outcome, and associations involving breastfeeding experience, obstetric characteristics, and psychosocial factors were treated as exploratory.

## 2. Materials and Methods

### 2.1. Sample and Population

This cross-sectional study was conducted at Etlik City Hospital, Ankara, Türkiye, between April and June 2025. The study was approved by the hospital’s Scientific Research Evaluation Ethics Committee (AEŞH-BADEK-2025-0122; 26 March 2025) and conducted in accordance with the Declaration of Helsinki. Written informed consent was obtained from all participants. No separate statistical analysis plan was prospectively documented or registered before data collection. Mothers attending routine pediatric well-child visits were recruited consecutively through nonprobability sampling and assessed once. Eligible participants were 4–24 weeks postpartum, literate in Turkish, and had delivered a singleton infant at 36–42 weeks of gestation. The lower postpartum limit was based on the validated use of the MAI from week 4 onward. Exclusion criteria included refusal of consent, multiple pregnancy, diagnosed psychotic-spectrum disorder, substance- or alcohol-use disorder, intellectual disability, and severe chronic systemic illness potentially compromising the validity of study assessments. The latter included severe cardiac, renal, or hepatic disease, active malignancy, and neurological disorders associated with substantial functional impairment. Eligibility was assessed during face-to-face interviews based on previously diagnosed conditions reported by the mothers and their potential interference with study assessments. No standardized assessment of disease severity or functional impairment was performed. Of 230 eligible mothers who received questionnaires, 212 completed them, while 18 returned entirely blank forms (response rate, 92.2%).

An a priori G*Power 3.1 calculation for the overall multiple regression model (R^2^ deviation from zero; f^2^ = 0.15; α = 0.05; 99% power; eight predictor parameters) required 211 participants. The final models each included seven predictor parameters, corresponding to 203 participants under the same assumptions; 212 mothers were analyzed. As the original calculation addressed overall model fit rather than the primary EPDS coefficient, a supplementary sample-size sensitivity analysis was performed. Under conventional linear regression assumptions, the available sample provided approximately 80% power to detect a partial correlation of 0.19 (partial f^2^ ≈ 0.0374; two-sided α = 0.05). This calculation was not part of the original sample-size planning and was not based on the observed effect.

### 2.2. Data Collection Method and Tools

Data were collected using a three-part questionnaire comprising sociodemographic and obstetric information [12,27], the EPDS, and the MAI. Pregnancy planning, spousal support, birth experience, breastfeeding experience, and other perinatal characteristics were assessed using investigator-developed, nonvalidated single-item questions. Sociodemographic and obstetric information was collected through face-to-face interviews, while participants completed the EPDS and MAI themselves. Data collection took approximately 15–20 min.

### 2.3. Edinburgh Postnatal Depression Scale

Postpartum depressive symptoms were assessed with the EPDS, a ten-item self-report screen covering the preceding seven days. Items are scored from 0 (“never”) to 3 (“most of the time”), giving a total between 0 and 30, with higher scores indicating greater symptom severity [28]. In the Turkish validation study, a cutoff of 12.5 gave the best balance of sensitivity (75.5%) and specificity (71.5%); mothers scoring ≥13 were therefore classified as screening positive for probable PPD [29].

### 2.4. Maternal Attachment Inventory

MIB was assessed with the MAI, a 26-item self-report scale developed by Muller [30]; its Turkish adaptation and psychometric validation were carried out by Kavlak and Şirin [31]. Items are scored from 4 (“always”) to 1 (“never”), giving a total between 26 and 104, with higher scores indicating stronger bonding.

### 2.5. Statistical Analysis

Continuous variables were summarized as mean ± standard deviation or median (interquartile range), and categorical variables as *n* (%). Bayesian Pearson correlation assessed the unadjusted EPDS–MAI association, while Bayesian Kendall’s τ-b assessed exploratory comparisons using the specified categorical contrasts and ordinal breastfeeding-initiation time among breastfeeding mothers (*n* = 200). Analyses used the jsq default stretched-beta prior (width 1) for Pearson’s ρ and corresponding Kendall prior [32,33]. BF_10_ expressed evidence for the alternative relative to the null under these priors, not a *p*-value or posterior probability. Frequentist regression provided adjusted estimates, 95% confidence intervals, and *p*-values; these inferential measures were interpreted separately. The corresponding two-sided frequentist Kendall’s τ-b *p*-values were jointly adjusted for 69 exploratory comparisons using the Benjamini–Hochberg procedure (Supplementary Table S1).

Covariates were selected for clinical and conceptual relevance rather than univariable significance. The EPDS model included pregnancy planning, spousal support during pregnancy and infant care, breastfeeding experience (joyful as reference), previous PPD, and infant age. The primary MAI model used linear regression with HC3 robust standard errors to estimate the EPDS association, adjusting for pregnancy planning, infant-care spousal support, temporary mother–infant separation, birth experience, previous PPD, and infant age. Birth experience was considered a potential antecedent associated with both outcomes, although retrospective appraisal could reflect current mood. Breastfeeding experience was included as a concurrent EPDS correlate but excluded from the primary MAI model because it might be an intermediate or downstream factor of uncertain temporal order. Adjusted estimates therefore describe conditional associations, not direct causal effects.

The primary coefficient summarized the association in observed MAI points as a best linear approximation, not a constant slope across EPDS values. HC3 addresses potential heteroskedasticity but not score boundedness or ceiling concentration. Diagnostics assessed residuals, influence, multicollinearity, specification tests, quadratic and cubic EPDS terms, natural cubic splines, and fitted values against scale limits. Because 83 mothers (39.2%) scored the MAI maximum of 104, three sensitivity models were fitted. Right-censored Tobit assumes a normally distributed, homoskedastic latent outcome with a linear conditional mean, potentially unsuitable for a psychometric ceiling. Fractional logistic regression modeled (MAI–26)/78 on [0, 1], retaining upper-boundary values without latent censoring. Exploratory ordinal logistic regression used data-derived categories (≤98, 99–101, 102–103, 104), which were neither prespecified nor clinically validated; proportional odds were assessed. Tobit and fractional-logit average marginal effects were expressed on the observed MAI scale. All models used the same sample and did not constitute independent replications.

Probable-PPD groups were compared using the Mann–Whitney U test and Cliff’s δ, categorical proportions using Pearson’s χ^2^ test, and screening positivity using a Wilson 95% confidence interval. Further checks examined alternative covariate sets, sparse-category exclusions, HC3 inference for the EPDS model, and parity adjustment (previous live births, primiparity, or gravidity). The Cinelli–Hazlett framework [34] assessed unmeasured confounding under equal-strength assumptions and conventional OLS inference. Exploratory analyses assessed correlated spousal-support measures, postpartum intervals (4–8, 9–12, and 13–24 weeks), EPDS-by-infant-age interaction, and exclusion or adjustment for maternal age below 20 years. Frequentist tests were two-sided (*p* < 0.05). Analyses were performed using jamovi (versions 2.3.18 and 2.6.44; jsq, GAMLj, and Rj modules) and R (version 4.4.1).

## 3. Results

The study included 212 mothers (mean age, 28.4 ± 4.7 years; median infant age, 9 weeks [IQR, 6–13]); 41 (19.3%) screened positive for probable PPD (EPDS ≥13), 136 (64.2%) were primiparous, and 83 (39.2%) reached the MAI maximum of 104 (median, 102; IQR, 99–104; Table 1). Five mothers (2.4%) were younger than 20 years, including one (0.5%) younger than 19; all were included in the analyses. Compared with those aged ≥20 years (*n* = 207), mothers younger than 20 (*n* = 5) had mean EPDS scores of 4.6 ± 5.2 versus 8.2 ± 5.3 (Mann–Whitney *p* = 0.146), median MAI scores of 102 in both groups (*p* = 0.810), and screening positivity of 20.0% versus 19.3%. Joyful, stressful/painful/anxiety-provoking, and no breastfeeding were reported by 80.0%, 20.0%, and 0% versus 83.6%, 10.6%, and 5.8%, respectively. These comparisons were descriptive given the small younger subgroup. Neither exclusion of mothers younger than 20 years (B = −0.205; 95% CI, −0.263 to −0.146) nor adjustment for maternal age (B = −0.198; 95% CI, −0.257 to −0.139) materially changed the primary EPDS–MAI association.

Pregnancy, childbirth, and postpartum characteristics are summarized in Table 2.

EPDS and MAI scores were inversely correlated (Bayesian Pearson r = −0.430; Kendall’s τ = −0.347; both BF_10_ > 100; Table 3). Of ten correlations involving maternal or obstetric characteristics, seven yielded BF_01_ > 3, favoring the null under the specified prior, and three were inconclusive.

Exploratory associations are shown in Table 4. Spousal support during pregnancy and infant care was associated with probable PPD screening status (both BF_10_ > 100). Compared with joyful breastfeeding, stressful breastfeeding and no breastfeeding were associated with higher EPDS scores in unadjusted Bayesian analyses (BF_10_ = 14.39 and 64.99), but neither continuous-EPDS comparison survived FDR correction.

Of 69 exploratory comparisons, 17 had unadjusted *p* < 0.05; four remained significant after Benjamini–Hochberg correction (*q* < 0.05): no breastfeeding versus joyful breastfeeding (*q* = 0.012), spousal support in infant care (*q* = 0.014), breastfeeding status (*q* = 0.014), and spousal support during pregnancy (*q* = 0.047). All concerned probable PPD screening status (EPDS ≥13); none involving continuous EPDS or MAI scores survived correction. Supplementary Table S1 reports all *p*- and *q*-values. These findings remain exploratory.

The multivariable EPDS model was significant (*p* < 0.001; adjusted R^2^ = 0.097). Planned pregnancy was associated with lower EPDS scores under conventional OLS inference (B = −2.19; *p* = 0.044), but not HC3 inference (95% CI, −4.83 to 0.46; *p* = 0.105). Relative to joyful breastfeeding, stressful, painful, or anxiety-provoking breastfeeding (B = 2.63; HC3 95% CI, 0.43–4.84; *p* = 0.019) and no breastfeeding (B = 3.72; HC3 95% CI, 1.00–6.43; *p* = 0.008) were associated with higher EPDS scores. Neither spousal-support measure nor previous PPD was significant after adjustment; infant age was also nonsignificant (B = 0.02; *p* = 0.761). Diagnostics showed Breusch–Pagan *p* = 0.480, Ramsey RESET *p* = 0.782, Shapiro–Wilk *p* = 0.003, maximum Cook’s distance = 0.089, and maximum conventional VIF = 1.47 (single-degree-of-freedom terms).

Exploratory analyses showed little change in the antenatal-support coefficient after adjusting for breastfeeding experience but attenuation after including infant-care support; the two support measures were correlated (Kendall’s τ-b = 0.53). Consistent support during pregnancy and infant care (*n* = 189), compared with other patterns (*n* = 23), was associated with lower EPDS scores in an adjusted combined-support model (B = −2.76; HC3 95% CI, −5.00 to −0.52; *p* = 0.016).

In the primary MAI model, higher EPDS scores were associated with lower MAI scores (B = −0.204; HC3 SE = 0.030; 95% CI, −0.262 to −0.145; standardized β = −0.384; adjusted R^2^ = 0.188; *p* < 0.001). The right-censored Tobit coefficient was −0.321 (95% CI, −0.428 to −0.213), with an observed-scale average marginal effect of −0.206; the fractional-logit marginal effect was −0.200. The ordinal-model OR per five-point EPDS increase was 0.45 (95% CI, 0.34–0.58; Table 5). Other covariates, including infant age (B = −0.006; *p* = 0.825), were nonsignificant. Diagnostics yielded Breusch–Pagan *p* = 0.76, Ramsey RESET *p* = 0.55, Shapiro–Wilk *p* < 0.001, maximum Cook’s distance = 0.157, and maximum VIF = 1.10.

Functional-form analyses yielded mixed findings: quadratic and joint cubic EPDS terms were nonsignificant (*p* = 0.179 and 0.317), as was Ramsey RESET (*p* = 0.547). Natural cubic spline comparisons varied with basis complexity (df = 4: likelihood-ratio *p* = 0.408; df = 5: *p* = 0.011), with possible nonlinearity at sparsely represented EPDS extremes. Restricting analyses to EPDS ≤17 (*n* = 200) yielded *p* = 0.087 for the five-degree-of-freedom spline. Primary-model fitted values (97.85–103.47) were within the theoretical MAI range (26–104), although the upper 95% confidence limit for the estimated mean reached 104.41.

Mothers screening positive for probable PPD had lower median MAI scores (99 [IQR, 98–101 vs. 103 [IQR, 100–104]; Mann–Whitney *p* < 0.001; Cliff’s δ = −0.54), fewer maximum scores (9.8% vs. 46.2%; *p* < 0.001), and more scores ≤98 (36.6% vs. 12.9%; *p* = 0.001) than those screening negative.

**Figure 1:**
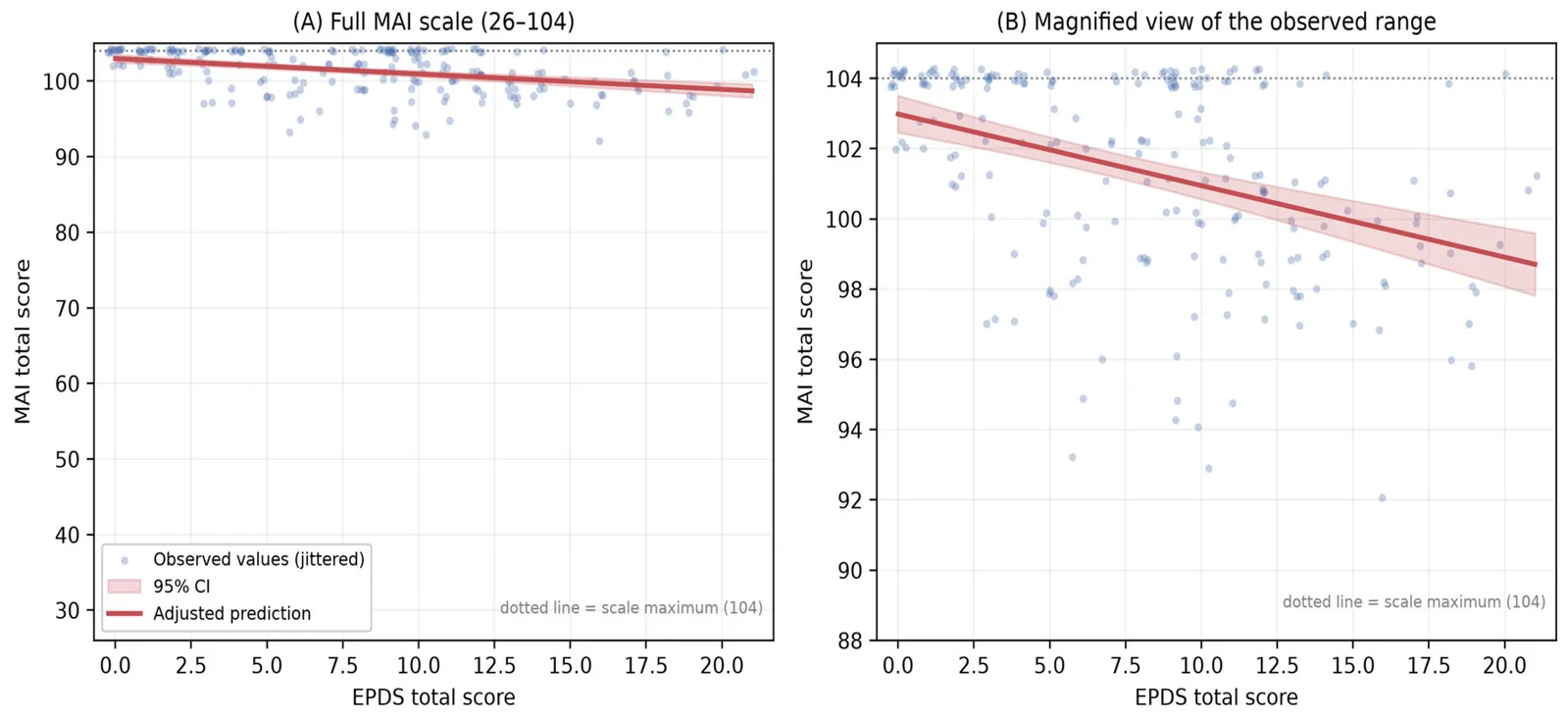
Association between EPDS and MAI scores (*n* = 212). Jittered points represent observations; the solid line and shading show adjusted linear-model predictions and HC3-based 95% confidence intervals. (**A**) Full MAI scale (26–104); (**B**) magnified observed range for visualization only. Effect magnitude should be interpreted using the full scale (Panel A). The model adjusted for pregnancy planning, spousal support in infant care, temporary mother–infant separation, birth experience, previous PPD, and infant age.

**Figure 2:**
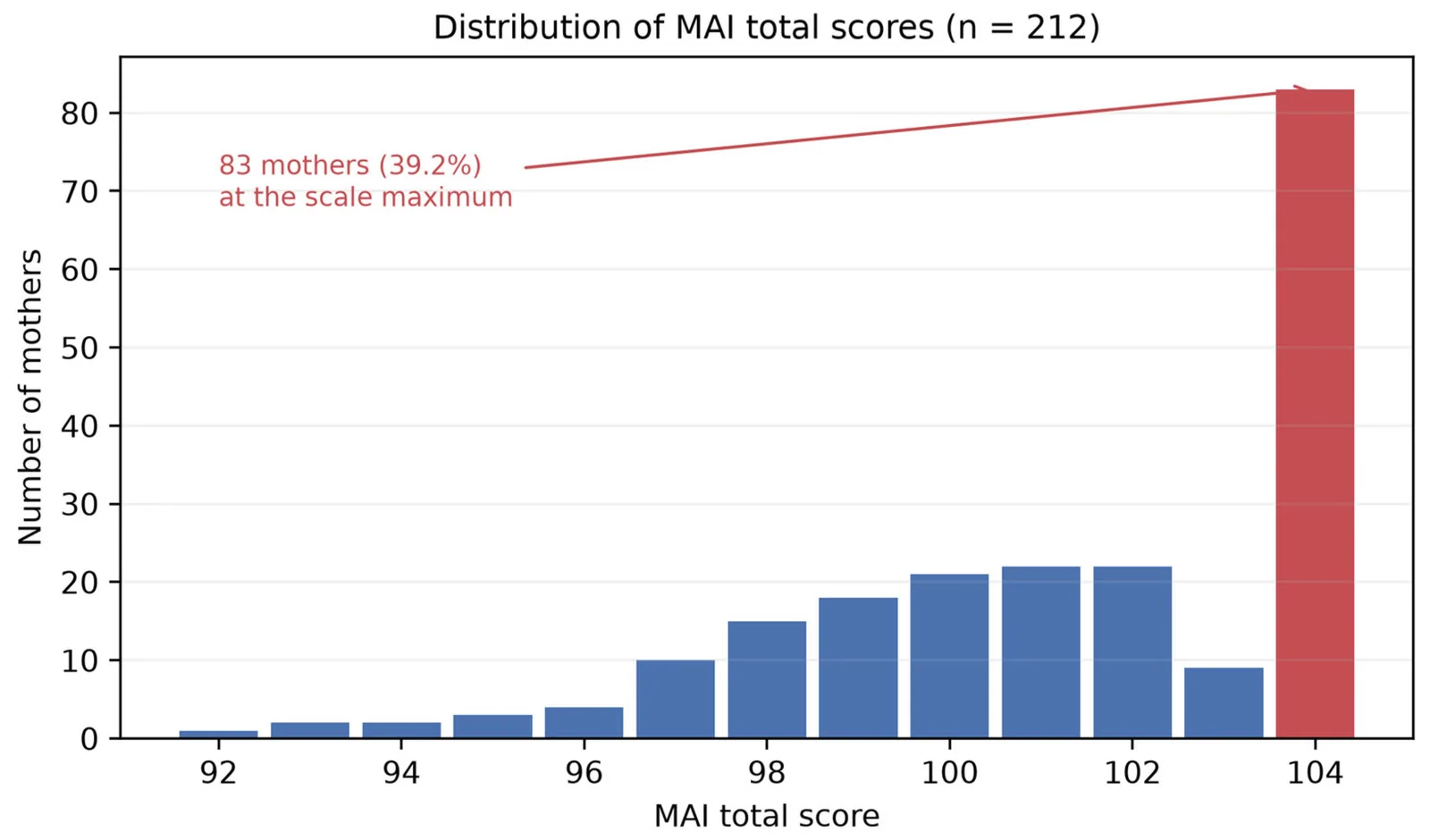
Distribution of MAI total scores among the 212 participants. The observed scores ranged from 92 to 104, with 83 mothers (39.2%) reaching the maximum score of 104.

After excluding sparse categories, linear EPDS coefficients ranged from −0.206 to −0.186 and Tobit coefficients from −0.325 to −0.307 (all *p* < 0.001). Alternative specifications yielded EPDS coefficients of −0.215 (adding breastfeeding experience), −0.209 (excluding birth experience), and −0.228 (unadjusted), versus −0.204 in the primary model. Figure 1 displays adjusted predictions; Figure 2 shows the MAI score distribution.

Parity-adjusted MAI models yielded EPDS coefficients of −0.2059 (95% CI, −0.264 to −0.148) with previous live births, −0.2051 (−0.263 to −0.147) with primiparity, and −0.2040 (−0.262 to −0.146) with gravidity, versus −0.2037 in the primary model; the additional terms were nonsignificant (*p* = 0.239, 0.319, and 0.847). In the Cinelli–Hazlett analysis, the partial R^2^ of EPDS with MAI was 0.146. Under equal-strength assumptions, an unmeasured confounder would need to explain 33.7% of residual variance in both EPDS and MAI to reduce the estimate to zero, or 24.0% of each to move the classical OLS confidence interval to include zero (α = 0.05).

Across postpartum intervals of 4–8 (*n* = 92), 9–12 (*n* = 60), and 13–24 weeks (*n* = 60), EPDS and MAI scores did not differ significantly (Kruskal–Wallis *p* = 0.265 and 0.262). Screening positivity was 19.6%, 18.3%, and 20.0%, respectively. Infant-age adjustment changed the EPDS–MAI coefficient from −0.2039 to −0.2037; the EPDS-by-infant-age interaction was nonsignificant (*p* = 0.222). Period-specific coefficients were −0.177, −0.187, and −0.234.

## 4. Discussion

In this cross-sectional study of 212 mothers assessed at 4–24 weeks postpartum, the principal finding was an inverse association between depressive symptom severity and MIB scores after adjustment, including for infant age. Screening positivity for probable PPD was 19.3%, close to the pooled estimate of 17.22% [4] and within the 14.7–26.5% range reported in Turkish studies [35,36]. Such comparisons are imprecise, however: recruitment setting, time since delivery, and socioeconomic circumstances shape which mothers are represented and how screening results should be read, and gender roles, socioeconomic differences, and access to care have all been proposed as sources of variation [37,38]. This figure denotes EPDS screening positivity rather than the prevalence of clinically diagnosed PPD. Secondary findings concerning breastfeeding, spousal support, and pregnancy planning were less robust and require cautious interpretation.

The adjusted EPDS–MAI association was similar across the primary linear model and the complementary Tobit, fractional-logit, and ordinal specifications. Because these models were fitted to the same 212 mothers, their agreement reflects consistency across analytical specifications rather than independent validation and does not overcome ceiling-related information loss. The direction of the association is also consistent with previous reports [39,40], including the earlier Turkish study conducted around two months postpartum [24]. In the primary analysis, five additional EPDS points corresponded to approximately one lower MAI point, a small observed-scale difference of uncertain clinical relevance. The clinical importance of this approximately one-point MAI difference remains uncertain, as the present study cannot establish whether a difference of this magnitude reflects meaningful bonding impairment. Mothers screening positive for probable PPD also had lower median bonding scores and were less likely to reach the MAI maximum, although that threshold is a screening classification rather than a clinical diagnosis. Tobit and fractional-logit marginal effects on the observed scale (−0.206 and −0.200) closely matched the linear estimate (−0.204), but exploratory splines suggested possible nonlinearity at sparsely represented EPDS extremes; the linear coefficient therefore summarizes an overall trend rather than a constant association across all EPDS levels. Reduced emotional availability, responsiveness, and mother–infant interaction quality are plausible explanations [17,41] but were not measured, and these pathways cannot be separated from mood-congruent self-reporting. Greater symptom severity may influence perceptions of bonding, yet bonding difficulties may equally contribute to distress; the cross-sectional data cannot distinguish them. Research on later child emotional and behavioral outcomes [42] provides context only; such outcomes were not examined here.

Before adjustment, spousal support was associated with fewer depressive symptoms, but neither antenatal nor infant-care support reached significance when both entered the adjusted EPDS model. Adding breastfeeding experience barely moved the antenatal coefficient, whereas adding infant-care support attenuated it, and the two support measures were correlated; an exploratory combined-support indicator was associated with lower EPDS scores. Shared information, confounding and mediation could each produce this pattern, and the present design cannot distinguish them; low multicollinearity establishes none of them. The measures were also nonvalidated single items, and the groups reporting no support were small. These findings should therefore be read alongside work on partner support, psychological well-being, parenting self-efficacy, and bonding [43,44,45,46], without presuming that improving support would necessarily reduce symptoms. The infant-sex association was confined to exploratory screening-status comparisons and should not be considered confirmatory.

In exploratory unadjusted analyses, planned pregnancy, a positive birth experience and infant-care support were associated with higher MAI scores, and temporary separation with lower ones, but none remained statistically significant after adjustment. Birth experience may be linked to bonding through depressive symptoms or other factors, and longitudinal evidence suggests such mediation [39], although that pathway was not tested here. Non-significance after adjustment does not rule out relevant associations, particularly where maternal characteristics and perceptions may be interrelated. These contrasts nonetheless carry less evidential weight than the primary EPDS–MAI estimate.

With only six mothers reporting previous PPD, this estimate is too imprecise to support a conclusion, and its lack of statistical significance after adjustment should not be read as evidence against the relevance of psychiatric history. The groups without spousal support were similarly small (*n* = 14 during pregnancy; *n* = 18 in infant care), though excluding these sparse categories did not materially alter the primary EPDS–MAI coefficient. The wider literature gives reason to keep psychiatric history in view: depression history has been related to postpartum symptoms and bonding [12], postpartum psychological difficulties to later child outcomes [47], and depressive symptoms have been reported to persist across early postpartum assessments even after lifetime depression history is taken into account [48]. Planned pregnancy was associated with lower EPDS scores under conventional standard errors but not under HC3 inference; with 26 unplanned pregnancies, this estimate also remains uncertain despite previous findings [49,50].

Stressful, painful or anxiety-provoking breastfeeding and not breastfeeding were both associated with higher adjusted EPDS scores than a joyful experience, whereas associations with MAI were less evident. Two considerations limit interpretation. The measures were single nonvalidated items, and the no-breastfeeding group was small (*n* = 12; B = 3.72; HC3 95% CI, 1.00–6.43). More importantly, breastfeeding experience and depressive symptoms were reported at the same visit, so mood-congruent appraisal cannot be ruled out and the temporal order of any difficulties was never observed; the literature accordingly describes the association as potentially bidirectional [17,51,52,53], while evidence implicating oxytocin-related mechanisms in PPD remains inconclusive [54]. What the present data cannot establish is whether breastfeeding-focused interventions would alter symptoms or bonding. A further qualification concerns the assessment window, since depressive symptoms and bonding may evolve across 4–24 weeks postpartum. EPDS and MAI scores did not differ significantly across the 4–8, 9–12 and 13–24-week intervals, adjustment for infant age barely changed the primary coefficient, and no clear EPDS-by-age interaction emerged. Nonsignificant differences do not, however, establish equivalence across stages, and a single assessment per mother cannot reveal within-person change.

Several limitations warrant consideration. The cross-sectional design precludes temporal or causal inference, including within-person change during the 4–24-week postpartum period. Consecutive nonprobability recruitment during well-child visits at one hospital, exclusion of births before 36 weeks and multiple pregnancies, and a relatively advantaged sample (54.3% university-educated; 39.2% with household income above USD 1200) restrict generalizability; only five mothers were younger than 20 years, one younger than 19, preventing meaningful conclusions about adolescents. Selection bias may have affected both screening positivity and the EPDS–MAI association in an unknown direction, and of 230 eligible mothers the 18 who returned blank questionnaires would underestimate screening positivity if more symptomatic mothers were less likely to respond. Concurrent self-report EPDS and MAI measurements may share affective content and introduce common-method, social-desirability or mood-congruent bias, and EPDS screening does not establish a clinical PPD diagnosis. The pronounced MAI ceiling effect (39.2%) limits discrimination at higher scores and complicates statistical modeling. Although alternative models yielded similar estimates, they neither provide independent validation nor recover information lost at the scale maximum. Exploratory splines suggested possible nonlinearity at sparsely represented EPDS extremes. Moreover, Tobit’s latent-censoring assumption may not adequately represent the psychometric ceiling, and the ordinal cutoffs were data-derived rather than validated. Pregnancy planning, spousal support, birth and breastfeeding experiences relied on nonvalidated single items reported 4–24 weeks postpartum, so recall bias may arise and temporal ordering remains uncertain, and small groups limit precision (nonbreastfeeding *n* = 12; unplanned pregnancy *n* = 26; previous PPD *n* = 6; absent spousal support during pregnancy *n* = 14 or infant care *n* = 18). Delivery mode, independently assessed anxiety, sleep, infant temperament, psychiatric treatment, intimate-partner violence and detailed socioeconomic measures were unavailable; parity-adjusted models and quantitative sensitivity analyses did not materially change the primary estimate, but residual confounding remains possible. Chronic-illness eligibility relied on maternal reports without standardized severity assessment, and the original sample-size calculation concerned overall model fit rather than the primary EPDS coefficient. Finally, of 69 exploratory comparisons examined, only four screening-status associations remained significant after false-discovery-rate correction, including overlapping breastfeeding contrasts; these secondary findings require independent confirmation.

## 5. Conclusions

In this single-center, cross-sectional sample of mothers assessed at 4–24 weeks postpartum, greater depressive symptom severity was associated with lower mother–infant bonding scores. This association persisted after multivariable adjustment and was consistent across complementary model specifications, although the absolute difference in MAI scores was small and its clinical significance remains uncertain. The 19.3% screening-positive proportion reflects probable PPD based on EPDS screening rather than clinically diagnosed depression. The cross-sectional design precludes causal inference, and longitudinal studies are needed to clarify the temporal relationship and clinical implications of these findings.

## Figures and Tables

**Table 1:** Sociodemographic and clinical characteristics of participants (*n* = 212).

Maternal Age (Years)	28.4 ± 4.7 (Range, 18–45)
Maternal age group, *n* (%)	
18–19 years	5 (2.4%)
20–24 years	38 (17.9%)
25–29 years	84 (39.6%)
30–34 years	61 (28.8%)
35–39 years	21 (9.9%)
40–45 years	3 (1.4%)
Infant age (weeks)	9 (6–13) (range, 4–24)
Educational level, *n* (%)	
Primary education	31 (14.6%)
Secondary education	66 (31.1%)
University degree	115 (54.3%)
Employment status, *n* (%)	
Not employed	117 (55.2%)
Employed	95 (44.8%)
Monthly household income, *n* (%)	
<600 USD	31 (14.6%)
600–1200 USD	98 (46.2%)
>1200 USD	83 (39.2%)
Family type, *n* (%)	
Nuclear family	194 (91.5%)
Extended family	18 (8.5%)
History of psychiatric illness, *n* (%)	
Yes	12 (5.7%)
No	200 (94.3%)
History of postpartum depression, *n* (%)	
Yes	6 (2.8%)
No	206 (97.2%)
EPDS total score	8.1 ± 5.3; median 9 (IQR 3–12); range 0–21
Screened positive for probable PPD (EPDS ≥13), *n* (%)	41 (19.3%; 95% CI 14.6–25.2)
Maternal Attachment Inventory	102 (99–104); range 92–104

Data are presented as *n* (%), mean ± SD, median (IQR), or range, as indicated. The 95% CI for probable PPD screening positivity was calculated using the Wilson method. EPDS, Edinburgh Postnatal Depression Scale; PPD, postpartum depression; MAI, Maternal Attachment Inventory; SD, standard deviation; IQR, interquartile range; CI, confidence interval; USD, United States dollars.

**Table 2:** Maternal Experiences During Pregnancy, Childbirth, and the Postpartum Period.

**Planned pregnancy**	
Yes	186 (87.7%)
No	26 (12.3%)
**Spousal support during pregnancy**	
Yes	198 (93.4%)
No	14 (6.6%)
**Illness during pregnancy**	
Yes	24 (11.3%)
No	188 (88.7%)
**History of miscarriage**	
Yes	45 (21.2%)
No	167 (78.8%)
**Birth experience**	
Joyful	94 (44.3%)
Stressful/Painful/Anxiety-provoking	118 (55.7%)
**Desired spousal presence at birth**	
Yes	178 (84.0%)
No	34 (16.0%)
**Spousal support in infant care**	
Yes	194 (91.5%)
No	18 (8.5%)
**External support in infant care**	
Yes	189 (89.2%)
No	23 (10.8%)
**Source of external support**	
Own family	170 (80.2%)
Spouse’s family	18 (8.5%)
Paid caregiver	1 (0.5%)
No support received	23 (10.8%)
**Illness during/after childbirth**	
Yes	12 (5.7%)
No	200 (94.3%)
**Temporary separation from infant**	
Yes	24 (11.3%)
No	188 (88.7%)
**Infant’s sex**	
Female	109 (51.4%)
Male	103 (48.6%)
**Mother’s infant-sex preference**	
No preference	127 (59.9%)
Female	68 (32.1%)
Male	17 (8.0%)
**Father’s infant-sex preference**	
No preference	120 (56.6%)
Female	63 (29.7%)
Male	29 (13.7%)
**Time to first breastfeeding**	
Immediately	132 (62.3%)
1–2 h	48 (22.6%)
3–4 h	10 (4.7%)
>4 h	10 (4.7%)
Did not breastfeed	12 (5.7%)
**Breastfeeding experience**	
Joyful	177 (83.5%)
Stressful/Painful/Anxiety-provoking	23 (10.8%)
Did not breastfeed	12 (5.7%)

Data are presented as *n* (%). All percentages were calculated using the total sample (*n* = 212), including those for sources of external support and timing of breastfeeding initiation.

**Table 3:** Bayesian Pearson Correlations of EPDS and MAI Scores with Selected Maternal and Obstetric Characteristics.

Variable	EPDS r	EPDS BF_10_	MAI r	MAI BF_10_
MAI score	−0.430	>100	—	—
Maternal age (years)	0.122	0.408	−0.150	0.924
Infant age (weeks)	0.033	0.096	−0.027	0.093
Number of pregnancies	0.045	0.106	−0.073	0.150
Number of miscarriages	0.028	0.093	−0.003	0.086
Number of live births	0.055	0.118	−0.136	0.605

EPDS, Edinburgh Postnatal Depression Scale; MAI, Maternal Attachment Inventory. Live-birth counts include the index birth. BF_10_ compares evidence for the alternative versus the null under the specified prior (BF_01_ = 1/BF_10_); values below 1 favor the null without proving absence of association, whereas values near 1 are inconclusive. Bayes factors are not frequentist *p*-values.

**Table 4:** Exploratory Bayesian Kendall’s τ-b Associations with EPDS, MAI, and Probable PPD Screening Status.

	EPDS		MAI		EPDS ≥ 13	
Variable (Coding)	**τ-b**	**BF_10_**	**τ-b**	**BF_10_**	**τ-b**	**BF_10_**
Educational level (primary < secondary < university)	0.006	0.091	0.014	0.094	−0.030	0.111
Employment status (employed vs. not employed)	0.000	0.090	0.036	0.122	−0.057	0.192
Household income (low < middle < high)	−0.018	0.097	−0.017	0.096	−0.051	0.165
Family type (extended vs. nuclear)	0.002	0.090	0.064	0.232	0.022	0.101
History of miscarriage	−0.002	0.090	−0.024	0.103	−0.108	1.378
Planned pregnancy	−0.139	8.351	0.127	3.954	−0.108	1.381
Spousal support during pregnancy	−0.154	22.570	0.063	0.228	−0.206	>100
Illness during pregnancy	−0.045	0.144	0.031	0.113	−0.062	0.220
Desired spousal presence at birth	−0.043	0.138	0.053	0.174	0.051	0.166
Birth experience (joyful vs. stressful/painful/anxiety-provoking)	−0.139	8.042	0.147	13.487	−0.125	3.351
Spousal support in infant care	−0.147	13.481	0.124	3.195	−0.236	>100
External support in infant care	−0.044	0.142	0.092	0.644	−0.021	0.100
Illness during/after childbirth	0.016	0.096	−0.052	0.171	0.087	0.521
Temporary separation from infant	0.081	0.417	−0.125	3.546	0.089	0.569
Past psychiatric history	0.056	0.187	−0.101	0.974	0.035	0.120
History of postpartum depression	0.113	1.740	−0.139	7.995	0.060	0.211
Infant’s sex (male vs. female)	0.023	0.102	−0.009	0.091	0.145	12.397
Mother’s infant-sex preference (any vs. none)	0.055	0.180	−0.013	0.093	−0.059	0.205
Father’s infant-sex preference (any vs. none)	0.081	0.409	−0.022	0.101	−0.019	0.098
Time to first breastfeeding (ordinal, later initiation; breastfed mothers, *n* = 200)	0.034	0.119	−0.081	0.388	0.077	0.336
Breastfeeding experience: stressful/painful/anxiety-provoking vs. joyful (*n* = 200)	0.151	14.392	0.003	0.093	0.129	3.579
Breastfeeding experience: did not breastfeed vs. joyful (*n* = 189)	0.177	64.992	−0.108	1.049	0.273	>100
Breastfeeding status: did not breastfeed vs. breastfed (*n* = 212)	0.158	29.392	−0.104	1.127	0.242	>100

Exploratory unadjusted associations were assessed using Bayesian Kendall’s τ-b (jsq default stretched-beta prior, width 1); *n* = 212 unless otherwise indicated. Variable categories correspond to Table 1 and Table 2. Binary contrasts coded the first-listed category as 1 (yes = 1 for yes/no variables); ordinal variables followed the stated order. Multicategory nominal variables were assessed using separate binary contrasts. Sources of external support were descriptive only because of sparse categories; support availability was analyzed as a binary variable. Breastfeeding-experience contrasts used joyful breastfeeding as reference (*n* = 200 and *n* = 189), while breastfeeding status was analyzed separately (*n* = 212). BF_10_ indicates evidence for the alternative relative to the null under the specified prior, not a frequentist *p*-value. EPDS, Edinburgh Postnatal Depression Scale; MAI, Maternal Attachment Inventory; PPD, postpartum depression.

**Table 5:** Association between EPDS score and MAI score across model specifications (*n* = 212 unless stated).

Model	EPDS Estimate (95% CI)	*p*
**Linear regression (HC3 SE), observed MAI scale (primary)**	B = −0.204 (−0.262–−0.145)	<0.001
**Right-censored Tobit regression**	B = −0.321 (−0.428–−0.213)	<0.001
**Tobit, average marginal effect on observed MAI scale**	−0.206 (−0.272–−0.140)	—
**Fractional logistic regression, average marginal effect**	−0.200 (−0.258–−0.141)	—
**Ordinal logistic regression, OR per 5-point increase in EPDS**	OR = 0.45 (0.34–0.58)	<0.001

The primary model used linear regression with HC3 robust standard errors; Tobit, fractional-logit, and ordinal logistic models assessed sensitivity to the MAI ceiling effect. All models adjusted for infant age and the same covariates as the primary MAI model. EPDS, Edinburgh Postnatal Depression Scale; MAI, Maternal Attachment Inventory; CI, confidence interval; OR, odds ratio.

## Data Availability

The data supporting the findings of this study are available from the corresponding author upon reasonable request. The dataset is not publicly available due to ethical and privacy considerations related to the protection of participant confidentiality, as specific consent for the public deposition of individual-level data in an open-access repository was not obtained from the participants.

## Supplementary Materials

The following supporting information can be downloaded at: https://www.mdpi.com/article/10.3390/jcm15218677/s1, Table S1: All 69 exploratory unadjusted associations of sociodemographic, psychosocial and perinatal factors with EPDS score, MAI score and probable PPD screening status, with Bayes factors, p values and Benjamini–Hochberg false-discovery-rate q values.

## Abbreviations

**BF_10_**	Bayes factor in favor of the alternative hypothesis relative to the null hypothesis
**CI**	Confidence interval
**EPDS**	Edinburgh Postnatal Depression Scale
**HC3**	Heteroskedasticity-consistent type 3 estimator
**IQR**	Interquartile range
**MAI**	Maternal Attachment Inventory
**MIB**	Mother–infant bonding
**PPD**	Postpartum depression
**VIF**	Variance inflation factor
