# Supplementary material for: Postpartum Depressive Symptoms and Mother–Infant Bonding at 4–24 Weeks Postpartum: A Cross-Sectional Study of Psychosocial and Breastfeeding-Related Factors
Source: J Clin Med. 2026 Nov 6;15(21):8677. doi: 10.3390/jcm15218677 (PMC13764269; doi:10.3390/jcm15218677)
Supplement: Supplementary file 1 [file jcm-15-08677-s001.zip › jcm-4603467-supplementary.pdf]

Table S1. All 69 exploratory unadjusted associations of sociodemographic, psychosocial and perinatal factors with EPDS score, MAI score and probable PPD screening status, with Bayes factors, p values and Benjamini–Hochberg false-discovery-rate q values.

No.	Variable (coding)	Outcome	n	Kendall's τ-b	BF_{10}	p value	q value (BH-FDR)	Survives FDR q<0.05
1	Educational level (primary < secondary < university)	EPDS score	212	0.006	0.091	0.912	0.968	No
2	Educational level (primary < secondary < university)	MAI score	212	0.014	0.094	0.816	0.909	No
3	Educational level (primary < secondary < university)	Probable PPD (EPDS ≥ 13)	212	−0.030	0.111	0.654	0.868	No
4	Employment status (employed vs. not employed)	EPDS score	212	0.000	0.090	0.999	0.999	No
5	Employment status (employed vs. not employed)	MAI score	212	0.036	0.122	0.551	0.786	No
6	Employment status (employed vs. not employed)	Probable PPD (EPDS ≥ 13)	212	−0.057	0.192	0.408	0.670	No
7	Household income (low < middle < high)	EPDS score	212	−0.018	0.097	0.749	0.884	No
8	Household income (low < middle < high)	MAI score	212	−0.017	0.096	0.772	0.884	No
9	Household income (low < middle < high)	Probable PPD (EPDS ≥ 13)	212	−0.051	0.165	0.438	0.674	No
10	Family type (extended vs. nuclear)	EPDS score	212	0.002	0.090	0.970	0.984	No
11	Family type (extended vs. nuclear)	MAI score	212	0.064	0.232	0.295	0.608	No
12	Family type (extended vs. nuclear)	Probable PPD (EPDS ≥ 13)	212	0.022	0.101	0.747	0.884	No
13	History of miscarriage	EPDS score	212	−0.002	0.090	0.967	0.984	No
14	History of miscarriage	MAI score	212	−0.024	0.103	0.695	0.884	No
15	History of miscarriage	Probable PPD (EPDS ≥ 13)	212	−0.108	1.378	0.116	0.321	No
16	Planned pregnancy	EPDS score	212	−0.139	8.350	0.016	0.094	No
17	Planned pregnancy	MAI score	212	0.127	3.954	0.036	0.167	No
18	Planned pregnancy	Probable PPD (EPDS ≥ 13)	212	−0.108	1.381	0.116	0.321	No
19	Spousal support during pregnancy	EPDS score	212	−0.154	22.570	0.008	0.077	No
20	Spousal support during pregnancy	MAI score	212	0.063	0.228	0.300	0.608	No
21	Spousal support during pregnancy	Probable PPD (EPDS ≥ 13)	212	−0.206	1,872	0.003	0.047	**Yes**
22	Illness during pregnancy	EPDS score	212	−0.045	0.144	0.438	0.674	No
23	Illness during pregnancy	MAI score	212	0.031	0.113	0.610	0.826	No
24	Illness during pregnancy	Probable PPD (EPDS ≥ 13)	212	−0.062	0.220	0.369	0.655	No
25	Desired spousal presence at birth	EPDS score	212	−0.043	0.138	0.459	0.674	No
26	Desired spousal presence at birth	MAI score	212	0.053	0.173	0.383	0.655	No
27	Desired spousal presence at birth	Probable PPD (EPDS ≥ 13)	212	0.051	0.166	0.456	0.674	No
28	Birth experience (joyful vs. stressful/painful/anxiety-provoking)	EPDS score	212	−0.139	8.042	0.016	0.094	No
29	Birth experience (joyful vs. stressful/painful/anxiety-provoking)	MAI score	212	0.147	13.487	0.016	0.094	No
30	Birth experience (joyful vs. stressful/painful/anxiety-provoking)	Probable PPD (EPDS ≥ 13)	212	−0.125	3.351	0.071	0.243	No
31	Spousal support in infant care	EPDS score	212	−0.147	13.481	0.011	0.087	No
32	Spousal support in infant care	MAI score	212	0.124	3.195	0.042	0.171	No
33	Spousal support in infant care	Probable PPD (EPDS ≥ 13)	212	−0.236	41,747	<0.001	0.014	**Yes**
34	External support in infant care	EPDS score	212	−0.044	0.142	0.443	0.674	No
35	External support in infant care	MAI score	212	0.092	0.644	0.131	0.348	No
36	External support in infant care	Probable PPD (EPDS ≥ 13)	212	−0.021	0.100	0.758	0.884	No
37	Illness during/after childbirth	EPDS score	212	0.016	0.096	0.776	0.884	No
38	Illness during/after childbirth	MAI score	212	−0.052	0.171	0.389	0.655	No
39	Illness during/after childbirth	Probable PPD (EPDS ≥ 13)	212	0.087	0.521	0.207	0.462	No
40	Temporary separation from infant	EPDS score	212	0.081	0.417	0.161	0.403	No
41	Temporary separation from infant	MAI score	212	−0.125	3.546	0.039	0.169	No
42	Temporary separation from infant	Probable PPD (EPDS ≥ 13)	212	0.089	0.569	0.197	0.452	No
43	Past psychiatric history	EPDS score	212	0.056	0.187	0.333	0.655	No
44	Past psychiatric history	MAI score	212	−0.101	0.974	0.097	0.290	No

No.	Variable (coding)	Outcome	n	Kendall's τ-b	BF_{10}	p value	q value (BH-FDR)	Survives FDR q<0.05
45	Past psychiatric history	Probable PPD (EPDS ≥ 13)	212	0.035	0.120	0.610	0.826	No
46	History of postpartum depression	EPDS score	212	0.113	1.740	0.051	0.197	No
47	History of postpartum depression	MAI score	212	−0.139	7.995	0.023	0.120	No
48	History of postpartum depression	Probable PPD (EPDS ≥ 13)	212	0.060	0.211	0.380	0.655	No
49	Infant's sex (male vs. female)	EPDS score	212	0.023	0.102	0.690	0.884	No
50	Infant's sex (male vs. female)	MAI score	212	−0.009	0.091	0.888	0.957	No
51	Infant's sex (male vs. female)	Probable PPD (EPDS ≥ 13)	212	0.145	12.397	0.035	0.167	No
52	Mother's infant-sex preference (any vs. none)	EPDS score	212	0.055	0.180	0.346	0.655	No
53	Mother's infant-sex preference (any vs. none)	MAI score	212	−0.013	0.093	0.835	0.915	No
54	Mother's infant-sex preference (any vs. none)	Probable PPD (EPDS ≥ 13)	212	−0.059	0.205	0.388	0.655	No
55	Father's infant-sex preference (any vs. none)	EPDS score	212	0.081	0.409	0.164	0.403	No
56	Father's infant-sex preference (any vs. none)	MAI score	212	−0.022	0.101	0.717	0.884	No
57	Father's infant-sex preference (any vs. none)	Probable PPD (EPDS ≥ 13)	212	−0.019	0.098	0.781	0.884	No
58	Time to first breastfeeding (ordinal, later initiation; breastfed mothers)	EPDS score	200	0.034	0.119	0.558	0.786	No
59	Time to first breastfeeding (ordinal, later initiation; breastfed mothers)	MAI score	200	−0.081	0.388	0.182	0.433	No
60	Time to first breastfeeding (ordinal, later initiation; breastfed mothers)	Probable PPD (EPDS ≥ 13)	200	0.077	0.336	0.262	0.566	No
61	Breastfeeding experience: stressful/painful/anxiety-provoking vs. joyful	EPDS score	200	0.151	14.392	0.011	0.087	No
62	Breastfeeding experience: stressful/painful/anxiety-provoking vs. joyful	MAI score	200	0.003	0.093	0.956	0.984	No
63	Breastfeeding experience: stressful/painful/anxiety-provoking vs. joyful	Probable PPD (EPDS ≥ 13)	200	0.129	3.579	0.069	0.243	No
64	Breastfeeding experience: did not breastfeed vs. joyful	EPDS score	189	0.177	64.992	0.004	0.053	No
65	Breastfeeding experience: did not breastfeed vs. joyful	MAI score	189	−0.108	1.049	0.095	0.290	No
66	Breastfeeding experience: did not breastfeed vs. joyful	Probable PPD (EPDS ≥ 13)	189	0.273	523,063	<0.001	0.012	**Yes**
67	Breastfeeding status: did not breastfeed vs. breastfed	EPDS score	212	0.158	29.392	0.006	0.074	No
68	Breastfeeding status: did not breastfeed vs. breastfed	MAI score	212	−0.104	1.127	0.087	0.286	No
69	Breastfeeding status: did not breastfeed vs. breastfed	Probable PPD (EPDS ≥ 13)	212	0.242	76,038	<0.001	0.014	**Yes**

Associations were summarized using Kendall's τ-b. Two-sided frequentist p-values were adjusted jointly across all 69 comparisons using the Benjamini–Hochberg false-discovery-rate procedure; q-values were computed from unrounded p-values. BF_{10} values were obtained separately from Bayesian Kendall's τ-b analyses using the jsq default stretched-beta prior (width 1) and compare the alternative with the null hypothesis. n = 212 unless otherwise indicated. For binary contrasts, the first-named category was coded 1; for yes/no variables, yes was coded 1. All four comparisons with q < 0.05 concerned probable PPD screening status rather than continuous EPDS or MAI scores. Because some breastfeeding contrasts overlap, these results should not be regarded as independent replications. EPDS, Edinburgh Postnatal Depression Scale; MAI, Maternal Attachment Inventory; PPD, postpartum depression.
